# Perceptions and Attitudes of Muslims in Israel When Using Porcine-Based Organs in Transplantation Procedures

**DOI:** 10.1007/s10943-025-02327-1

**Published:** 2025-05-13

**Authors:** Mahdi Tarabeih, Wasef Na’amnih

**Affiliations:** 1 School of Nursing Sciences, The Academic College of Tel-Aviv-Yaffa, Rabenu Yerucham St, P.O.B 8401, 64044 Tel Aviv, Israel; 2 Department of Epidemiology and Preventive Medicine, School of Public Health, Sackler Faculty of Medicine, Tel Aviv University, 69978 Tel Aviv, Israel

**Keywords:** Muslim, Porcine-derived products, Religion, Xenotransplantation

## Abstract

Porcine-derived organ transplants may arouse resistance due to Muslim religious prohibitions against consuming pork products. However, according to Sharia, pork is permitted for life-saving purposes. We performed a cross-sectional study among 884 Israeli Muslims aged 18–81 years with different levels of religious observance, socioeconomic status, and education to assess the knowledge and attitudes of Muslims regarding porcine xenotransplantation according to the Islamic perspective. We used an online questionnaire that was posted on social media. Our findings demonstrated that Muslims were unaware of what is permitted by Islam. Thus, we offer recommendations for improving the informed consent process with Muslim patients. We believe that the findings of our study will be used as an evidence-based source for deliberations among physicians, religious leaders, and theologians to promote knowledge in members of their religions and, no less importantly, the cultural competence of medical and nursing staff with respect for the autonomy of any patient.

## Introduction

Patients’ religious beliefs have strongly influenced medical decisions and choice of treatment. The ingestion of porcine products is strictly prohibited in Islam; therefore, being treated with porcine products can lead to religious distress. Thus, the administration of porcine-based medical treatment could be perceived as an insensitive practice (Ali et al., 2022).

There are currently 1.9 billion Muslims in the world, with Sunnis (87%−90%) and Shiites (10%−13%) being the two main sects. These two sects represent 23% of the world’s estimated population of 8 billion in 2022 (Pew Research Center, 2022). Many live in Western countries and constitute an ethnic minority whose beliefs and values must be considered when undergoing medical treatment (Paris et al., 2018).

In light of the growing importance worldwide of patient-centered care, we focused on the need to adapt medical care to Muslim patients when implants are derived from pigs, an animal considered impure according to Islam. There is scarce empirical data examining Muslim patients’ knowledge and attitudes towards using these products (Wachholtz, 2000).

Worldwide, end-stage organ failure rates have continued rising, while organ donation rates have not kept pace (Bastani, 2020; Cantarovich, 2023; Chen et al., 2024; Wachholtz, 2000). Concurrently, scientific knowledge and experience with xenotransplantation (XTx) continue to advance, thus, portending the possibility that immunological advances may soon overcome physiological and safety-related barriers to the clinical adoption of XTx as a treatment option (Sautermeister, 2015).

This development raises many challenging theological-ethical and social issues that should be considered, both for society and the potential recipient. Public opinion polls have shown that the perception and willingness to consider XTx is generally positive, although religious beliefs’ influence is less straightforward (Sautermeister, 2015). A full understanding of the beliefs and practices of the Islamic tradition is necessary to fully prepare for XTx clinical trials and porcine organ transplantation.

### Xenotransplantation

Xenotransplantation is the transplantation of cells, tissues, or organs from one species to another. One type of xenotransplantation is the transplantation of organs from other species into humans. The transplanted organ or tissue is called a xenograft. (Cooper et al., 2015; Kim & Hara, 2015). The initiative to transplant organs from other species into humans has arisen due to the global shortage of human organs for transplantation, with patients occasionally dying as they wait for an organ donation (Bastani, 2020; Cantarovich, 2023; Chen et al., 2024; Cooper et al., 2015; Mantovani et al., 2023; Mohiuddin et al., 2015).

Advances in understanding the organ rejection process have led scientists to become proficient in organ transplantation from other species. Because of the similarity between certain internal organs of humans and pigs, the process has been mainly discussed in the context of transplanting porcine organs into humans with the hope of solving the shortage of organs for transplantation (Cowan et al., 2014; Kim & Hara, 2015; Mohiuddin et al., 2015).

Pigs are the most favored animal for xenotransplantation for several reasons. First, their organs are similar in size to that of human beings and can be modified genetically for transplanting with less chance of rejection. Second, pigs are relatively easy to breed and produce large litters (8–12 offspring) several times in one year. Third, the pig is also preferred over non-human primates which are closer genetically to humans. This is economically practical because of the substantial research time and money already invested in developing a genetically modified pig that would serve as a viable model for transplantation. Chimpanos and other monkey species are classed officially as endangered species and are thus less favored. Thus, the pig is a more natural choice in terms of availability and cost (Ali et al., 2023).

There have been several successful attempts to transplant a pig organ into a human being. Several patients who needed a liver transplant were treated with a temporary pig liver transplant. Certain patients with Parkinson’s disease who were treated with pig neurons experienced significant improvement (Fink et al., 2000).

### Xenotransplantation Experimentation: Progress and Setbacks

An important breakthrough in the field of porcine organ transplants was reported when American researchers performed a successful transplant of a heart from a genetically modified pig (Wang et al., 2022). This type of transplantation significantly promoted the prospects of transgenic animal organ transplants, thus reducing the risk of future rejection of the transplanted organ. The breakthrough was reported just months after the publication of groundbreaking clinical trials at the NYU Langone Transplant Institute on transplanting pig-derived kidneys into brain-dead humans (Wang et al., 2022).

Robert Montgomery, MD, Director of the Institute of Langone Transplants at NYU performed a breakthrough procedure in 2023 (Locke et al., 2023; Montgomery et al., 2022; Porrett et al., 2022; Wang et al., 2022) of a kidney transplant taken from a pig with only one genetic change and without drugs or experimental devices. He claimed that this procedure can replace the function of a human kidney “and continue to function for more than a month without being rejected” (Montgomery et al., 2022).

Scientists at Harvard Medical School transplanted a genetically modified pig kidney into a living human, raising hopes for saving lives amid a lack of human kidney donors and reducing health disparities associated with organ failure and transplants. The patient, afflicted with type 2 diabetes and hypertension, received the kidney in March 2024 and survived for nearly two months. His death was not attributed to the transplant (Mallapaty, 2024).

Pigs are superior to other mammals when used for growing organs intended for transplants because they are easy to breed and raise, and their organs achieve the size of a human organ in only six months. For this reason, in recent years, pig heart valve transplants have been routinely performed in human heart valve replacement surgeries, especially in cases of heart valve disease leading to heart failure. Similarly successful have been experiments in transplanting fetal cells from the pancreas of pigs into type 1 diabetes patients—experiments that indicated effectiveness in renewing the body’s ability to produce insulin (Elisseeff et al., 2021; Wang et al., 2022; Xi et al., 2023).

Furthermore, over the past decade, several studies have been examining porcine skin grafts. In 2019, American researchers from the Massachusetts General Medical Center reported a breakthrough in the field, when they succeeded in transplanting skin tissues from transgenic pigs into burn victims (Elisseeff et al., 2021; Estrada et al., 2015; Wang et al., 2022).

During the COVID-19 epidemic, there have been lengthy waiting times for organ transplants worldwide. In Israel, according to the Ministry of Health, the number of people waiting for organ transplants increased by 10% from 1123 in 2019 to 1299 in 2022 (Pew Research Center, 2022).

Owing to the progress of clinical trials using products derived from pigs and the positive predictions for the future, it is crucial to examine the theological views of Islam on this issue, to investigate how its religious rulings relate to human health and the use of pig organs for medical purposes (Padela, 2013; Paris et al., 2018). Furthermore, there is little empirical data examining the knowledge and attitudes of Muslim patients towards the use of such devices.

### Islamic Bioethical View on Porcine Xenotransplantation

Islam is defined as a perfect or complete religion, encompassing all aspects of human life. Its precepts demonstrate care for its follower’s well-being in everyday life, especially in potentially harmful situations (Hamid & Mokhtar, 2019; Kader, 2021). A study offering an in-depth examination of the Islamic position on preserving life and good health showed that this is an important principle in the religion. Muslims are expected to maintain their physical health to fulfill their duty to God (Qureshi & Padela, 2016). According to the hadith,"God has sent the disease and the medicine, and He has appointed a medicine for every disease, so treat yourself with medicine, but do not use anything that is against the laws of Islamic Sharia” (Qureshi & Padela, 2016).

The Islamic prohibition against consuming products taken from a pig is brought down in religious writings. The Qur’an teaches that “you are forbidden [the consumption] of carrion, blood, swine flesh… for these are impure” (Al-Qur’an, Sura Al-Ma’idah, 5:3; Sura Al-An’aam, 6:145). Because life is a gift of Allah, it is sacred and cherished; therefore, the individual is commanded to save a human life (Daar & Khitamy, 2001). The saving of a life takes precedence above all other religious duties (Hedayat & Pirzadeh, 2001). The Qur’an states that in a life-threatening situation, “…necessities overrule prohibitions” (Al-Qur’an, Surah Al-Baqarah, 2:173); accordingly, pork, which is specifically prohibited for consumption, is permitted under certain circumstances where no lawful alternative exists.

These ostensible leniencies are mentioned in the Qur’an in four different verses. clearly stating that eating the meat of pigs is forbidden. For example, “Forbidden to you (to eat): dead meat, blood, the flesh of swine, and that on which hath been invoked the name of other than Allah“ (Al-Qur’an, Sura Al Maeeda, 5:3). However, the scripture on this subject goes on to state in three additional places: “But if one is forced by necessity, without willful disobedience, nor transgressing due limits, then he is guiltless. For Allah is Oft-forgiving Most Merciful“ (Al-Qur’an, Sura Al-Baqarah, 2:173; Sura-Al-Anaam, 6:145; Sura Al-Nahl, 16:115). Islam permits the consumption of pigs, porcine-derived medication, and surgical products in life-threatening situations. This leniency, termed “Darurah”, is permissible only after all other alternatives have been exhausted: “Dire necessity renders the impermissible to be permissible” specifically, for lifesaving and health purposes (Padela et al., 2014; Shiwani, 2020).

### Sunni View on Xenotransplantation

Sunni lawmakers put forward a prioritized list of animals that can be used for xenotransplantation, which applies even when dire necessity is cited. This is due to two factors: the first is the strict prohibition against using prohibited substances (tadāwī bi al-muḥarramāt) even for medical treatment. The second is a general aversion to the pig derived from sacred texts (Ali et al., 2023). Other restrictive conditions apply in Islamic theology to using animals for human purposes, which require humans not to abuse the animal kingdom they are commanded to care for. Sunni scholars permit the use of xenograft in case of dire necessity out of the belief that xenotransplantation therapy is preferable to the use of a human donor with the attendant violation of human dignity. Accordingly, most Sunni jurists do not prohibit xenotransplantation from pigs when no other options exist and when the recommended therapy is effective, which is not always the case with xenotransplantation. The prioritized list of animals to be used is first, animals that are allowed to be eaten; then animals such as primates that are considered by scripture to be clean though not to be eaten, followed by ritually unclean animals, among them, the pig (Ali et al., 2023). Additional factors that inform Muslim decision-making in healthcare are the unknown risks of adverse immunological reactions to the treatment as well as concerns regarding transmitting cross-species viruses. However, despite the fatwas that may permit xenotransplantation, one must recognize the social considerations that prevent patients from accepting this solution. These include issues of self-image and fear of altered consciousness and self-experience. Because xenotransplantation may cause patients a sense of spiritual unease, porcine xenotransplantation should be regarded as a temporary or last-resort solution for the problem of organ failure (Ali et al., 2023; Veatch & Ross, 2015).

### Shia View on Xenotransplantation

Among Shia jurists, xenotransplantation has raised little controversy and is sanctioned by all the authorities underlying Shia jurisprudence, the Qur’an, tradition, reason, and consensus. All the fatwas by Shia jurists on the subject of transplantation have proclaimed its permissibility because it fulfills the basic criteria for permissibility. Prominent among these criteria are a dire necessity and not causing harm (Aramesh, 2023). These fatwas are accompanied by two conditions that must be met: first, there must be a definite medical necessity; second, they must not entail other prohibited acts. In these circumstances, the animals to be used do not necessarily have to be slaughtered according to Islamic ritual law, and no distinction is drawn between unclean and halal animals (Aramesh, 2023).

Under Islamic religious law, xenotransplantation from pig organs must be viewed from the perspective of the ethical status of the act and its purpose. An additional factor is how it will ultimately affect the patient and the wider implications of this practice for society as a whole (Paris et al., 2018). In their study, Paris and colleagues (2018) found that demographic factors influence people who refused or consented to treatment with porcine-derived medications and implants. These factors included religion, age, gender, and education. Moreover, a positive correlation was found between knowledge of religious law and positive attitudes regarding the use of organs taken from a pig for healthcare purposes. The severe shortage of human organs for transplantation renders an organ taken from a pig the only effective treatment for early-stage organ failure (Cooper, 2020; Cooper et al., 2018; Paris et al., 2018; Puga Yung et al., 2017).

### Aims and Objectives

Muslim patients may experience distress when the attending physician recommends treatment, a medicinal preparation, or the transplantation of an organ derived from pigs due to their fear of violating the religious prohibitions of Sharia. Nonetheless, there is still no empirical data that have examined the knowledge of Muslims regarding whether pig organ transplants are permitted, nor their approach to the question of whether the religious authorities should allow the use of these organs.

Our study aimed to fill this gap and described the findings of the first large-scale study conducted on this topic in Israel, where Muslims comprise 21% of the total population (Central Bureau of Statistics, 2022; Pew Research Center, 2022). The study sought to obtain answers specifically to the following research questions: (1) What is the level of knowledge among Muslims regarding the use of transplanting organs of porcine origin for medical purposes? (2) What is the Muslims’ attitude as to whether porcine organs should be permitted for transplantation? (3) Is the knowledge of Islam’s rulings on the use of transplants of porcine origin positively correlated with a favorable attitude?

## Methods

### Study Design and Population

We performed a cross-sectional study among Israeli Muslims aged 18–81 years with different levels of religious observance, socioeconomic status, and education. The inclusion criteria were age ≥ 18 years and readiness to participate. We used a snowball method and convenience sampling for our study. The online questionnaire was posted on social media to 938 Israeli Muslims; of those, 884 Israeli Muslims agreed to participate and complete the online questionnaire. The participants were informed of the subject matter of the study: medical uses of organs originating from pigs.

#### Study Tool and Data Collection

The online questionnaire was built by the authors and validated by five people who are experts in the field. It was then translated into English and Arabic languages. In addition, a pilot study was conducted on 40 participants to examine the internal reliability of the questionnaire with Cronbach’s alpha of 0.78 for all the questions in the questionnaire. The questionnaire comprised 3 parts: a) Demographic data: age, gender, education, level of religiosity, marital status, and number of children; b) The level of knowledge regarding porcine xenotransplantation according to the Islamic religion; c) Respondents were asked to express their opinion regarding porcine xenotransplantation according to the Islamic perspective. We deliberately included different types of organ transplants (heart, kidney, lung, pancreas, knee cartilage, and skin transplants), some of them lifesaving and some less so or not at all. We asked the respondents to rank the transplant according to the necessity and importance of the organ for saving a life or not.

The variables ‘knowledge’ and ‘opinion’ measures consisted of 6 corresponding items regarding porcine xenotransplantation according to the Islamic perspective with different Likert scales for ‘knowledge’ and ‘opinion’. The respondents were instructed to consider each medical organ transplant twice. Initially, they were asked to report their level of knowledge as to the degree of approval under Islamic law for any pig-based organ transplant. They answered using a 7-point Likert scale [(1) It is 100% forbidden, (2) It is almost always forbidden, (3) It is permitted infrequently, (4) It is permitted in exceptional cases, (5) It is always almost permitted, (6) It is totally 100% permitted (7) I don’t know]. Subsequently, they were asked to express their opinion as to what extent they believe that their religion should allow any transplant of a porcine-derived organ, using a 7-point Likert scale [(1) Total disagree, (2) Disagree somewhat, (3) Agree slightly, (4) Agree moderately, (5) Agree to a large extent, (6) Agree strongly, (7) Total agree] (Appendix A). Then, we built two variables, ‘knowledge’ and ‘opinion’ based on the calculation of the average of the answers to the six medical treatments of a porcine transplant.

### Statistical Methods Data Analysis

The Kolmogorov–Smirnov test was performed to examine the normal distribution. Variables in our study were distributed normally and presented with the means and standard deviations of each item, and Pearson zero-order correlations between each dyadic set of items. We used the paired-sample *t*-tests for continuous variables to assess the degree of statistical difference between the 6 sets of items from both measures (i.e., item 1 in ‘knowledge’ vs. item 1 in ‘opinion’). We also designed a path analysis model and mediation effect analyses using standardized regression coefficients to explain the attitude toward the use of porcine-derived materials for medical purposes. All statistical tests were performed using the Statistical Package for the Social Sciences (SPSS), version 27 (IBM, Armonk, New York, NY, USA), and P < 0.05 was considered statistically significant.

### Ethical approval

The study was approved by the appropriate institutional review board: approval number 2022 −1082. Respondents signed informed consent forms to participate in the study and to allow their data to be used by the study team. The respondents were assured that their data would be kept confidential.

## Results

In total, of 884 Israeli-Muslim participants aged 18–81 years (mean = 49.4, SD = 20.4), 427 (48.3%) were males, and 457 (51.7%) were females, 49.7% and 45.5% were academic (university education) and religious participants, respectively. The mean number of children was 4.43, SD = 2.84 (Table 1).

**Table 1:** Participant demographic characteristics

Variable	Category	*N*	%	*M*	*SD*	*R*
*Gender*	Male	427	48.3	–	–	-
	Female	457	51.7	–	–	–
*Education*	Not academic	439	49.7	–	–	–
	Academic	445	50.3	–	–	–
*Religiosity*	Religious	402	45.5	–	–	–
	Secular	482	54.5	–	–	–
*Marital Status*	Not in a relationship In a relationship	86 798	9.7 90.3	–	–	–
*Age*		–	–	49.41	20.43	18–81
*Number of children*		*-*	–	–	*2.84*	*0–13*

Abbreviations: *N* Frequency *%* Relative percent *M* = Mean *SD* Standard deviation *R* Range

The ‘knowledge’ and ‘opinion’ measures consisted of 6 corresponding items as detailed in the method section. Table 2 presents the means and standard deviations of each item, and zero-order correlations between each dyadic set of items. In addition, paired-sample *t*-tests assessed the degree of statistical difference between the 6 sets of items from both measures (i.e., item 1 in ‘knowledge’ vs. item 1 in ‘opinion’, item 2 in ‘knowledge’ vs. item 2 in ‘opinion’, and so forth). Table 2 indicates that Participants were more assured in their personal ‘opinion’ on items 1–3 than in their generic religious ‘knowledge’: (1) “Is it permissible according to the Islamic religion to transplant a heart or a heart valve taken from a pig into a patient suffering from a heart problem whose life is in danger?” (2) “Is it permissible according to the Islamic religion to perform a lung transplant in a patient suffering from severe obstructive pulmonary disease, using a lung taken from a pig?” (3) “Is it permissible according to the Islamic religion to perform a kidney transplant in a patient suffering from severe renal insufficiency using a kidney taken from a pig?”. However, participants were more assured in their ‘knowledge’ of items 4–6, than in their personal ‘opinion’: (4) “Is it permissible according to the Islamic religion to treat a patient who is in danger of dying from pancreatic insufficiency by performing a pancreas/pancreatic cell transplant taken from a pig?” (5) “Is it permissible according to the Islamic religion to implant in a patient’s knee cartilage taken from a pig to replace worn cartilage?” (6) “Is it permissible according to the Islamic religion to use skin tissue of a pig to perform a skin graft in a patient with serious burn injuries?”.

**Table 2:** Correlations for all knowledge and opinion items

	‘knowledge’ items		‘opinion’ items			
Item	*M*	*SD*	*M*	*SD*	*r*_p_	*t*-test
1. Is it permissible according to the Islamic religion to transplant a heart or a heart valve taken from a pig into a patient suffering from a heart problem whose life is in danger?	3.14	1.95	3.35	2.35	.96	8.48^***^
2. Is it permissible according to the Islamic religion to perform a lung transplant in a patient suffering from severe obstructive pulmonary disease, using a lung taken from a pig?	2.80	1.88	2.91	2.23	.96	4.51^***^
3. Is it permissible according to the Islamic religion to perform a kidney transplant in a patient suffering from severe renal insufficiency using a kidney taken from a pig?	2.91	1.94	2.98	2.29	.95	2.56^*^
4. Is it permissible according to the Islamic religion to treat a patient who is in danger of dying from pancreatic insufficiency by performing a pancreas/pancreatic cell transplant taken from a pig?	2.40	1.82	2.12	1.80	.94	12.19^***^
5. Is it permissible according to the Islamic religion to implant in a patient’s knee cartilage taken from a pig to replace worn cartilage?	2.22	1.74	1.71	1.42	.87	16.74^***^
6. Is it permissible according to the Islamic religion to use the skin tissue of a pig to perform a skin graft in a patient with serious burn injuries?	2.05	1.65	1.38	1.01	.79	17.11^***^

^*^P <.05, **P <.01, ***P <.001. Abbreviations: *M* Mean *SD* Standard deviation *r* = Pearson zero-order correlation coefficient (all correlations are significant at P <.001). Generally, the internal consistencies, as measured by Cronbach’s Alpha reliability coefficients, of the ‘knowledge’ and ‘opinion’ variables are above.90 for each variable

Furthermore, to enhance the understanding of the underlying factors predicting the changes in ‘opinion’, a correlation matrix was gleaned from the data (Table 3), as a basis for further analysis. The level of religiosity was associated with the knowledge and attitudes of Muslims on porcine xenotransplantation. This is indicated in Table 3 showing a significant positive correlation between the level of religiosity and ‘knowledge’ (r = 0.39, P > 0.01) and attitudes (r = 0.44, P > 0.001). We found that more religious individuals were generally more confident of their ‘knowledge’ regarding porcine xenotransplantation than the less religious (secular) participants​. This suggests that higher religiosity is associated with greater awareness of Islamic rulings on the subject. However, interestingly, the data also showed that more religious individuals were more likely to hold favorable personal opinions toward the use of porcine-derived organs for medical purposes. Thus, religiosity plays a significant role in shaping both the understanding and the personal beliefs of Muslims regarding the permissibility and acceptance of porcine xenotransplantation (Table 3).

**Table 3:** Correlations between demographic factors, knowledge and opinion items

	1	2	3	4	5	6	7	8
1. Age	–							
2. Gender	−.02	–						
3. Education	.06	−.01	–					
4. Religiosity	.02	.01	.01	–				
5. Marital status	.63^***^	-.13^***^	−.01	−.11^***^	–			
6. Number of children	.83^***^	.11^**^	-.19^***^	-.27^***^	.65^***^	–		
7. Knowledge	.62^***^	-.03	.21^***^	.39^**^	.22^***^	.44^***^	–	
8. Opinion	.75^***^	-.05	.19^***^	.44^***^	.43^***^	.52^***^	.93^***^	–

^*^P < .05, **P < .01, ***P < *.001*

**Fig. 1:**
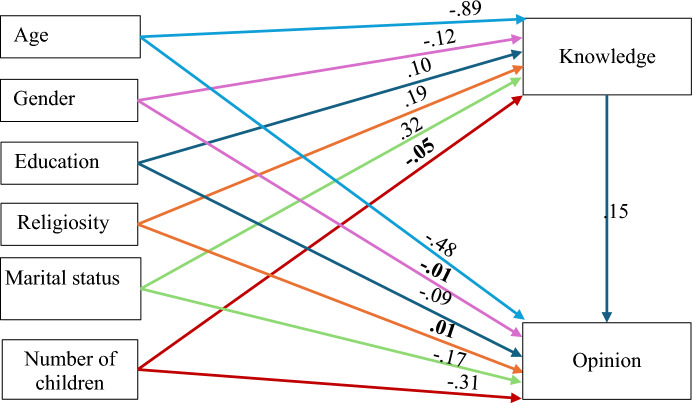
Mediation model path diagram with standardized regression coefficients. All paths are significant at P <.001, apart from bolded non-significant statistics

The preliminary results from Table 3 showed moderate-to-high and statistically significant correlations between demographic factors and both the ‘knowledge’ and the ‘opinion’ latent factors. After assessing these data, path analysis and structural equation modeling (SEM) were employed to explore a mediation model. In this model, the demographical parameters act as predictors for ‘knowledge’ as the mediator and ‘opinion’ as the criterion. The model has fit in the absolute sense; however, the path analysis bootstrapping method assessed the mediation effect (5,000 resamples, 95% bias-corrected confidence interval). Tables 4 and 5 and Fig. 1 indicate that most of the paths/associations were statistically significant: Age negatively correlates with ‘knowledge’ (β = − 0.89), and ‘opinion’ (β = − 0.48) regarding porcine xenotransplantation, P < 0.001. Men are more assured of their ‘knowledge’ (β =− 0.12) regarding porcine xenotransplantation than women, P < 0.001. Those with academic education are more assured of their ‘knowledge’ (β = 0.10) regarding porcine xenotransplantation than those without academic education P < 0.001. However, those without academic education are more assured of their ‘opinion’ (β = − 0.09) regarding porcine xenotransplantation than those with academic education P < 0.001. Religious and married individuals are more assured of their ‘knowledge’ (β = 0.19, and β = 0.32, respectively) regarding porcine xenotransplantation than less religious (i.e., secular) people P < 0.001. However, unmarried individuals are more assured of their ‘opinion’ (β = − 0.17) regarding porcine xenotransplantation than those who are married. Number of children negatively correlates with ‘opinion’ (β = − 0.31) regarding porcine xenotransplantation, P < 0.001. ‘knowledge’ positively correlates with ‘opinion’ (β = 0.15), regarding porcine xenotransplantation, P < 0.001 (Table 4 and Fig. 1). Moreover, most of the indirect effect was significant (i.e., the zero is outside the confidence interval’s limits). This renders ‘knowledge’ as a *partial mediator* between the set of predictors (age, gender, education, religiosity, marital status) and the criterion (i.e., ‘opinion’). However, ‘knowledge’ did not act as a mediator between the number of children and the ‘opinion’, P = 0.330 (Table 5).

**Table 4:** Path analysis results using standardized regression coefficients and difference tests

Path			b	*SE*	*t-test*	*P value*
Age	→	Knowledge	−.89	0.00	−22.22	<.001
Gender	→	Knowledge	−.12	0.06	−6.40	<.001
Education	→	Knowledge	.10	0.07	4.68	<.001
Religiosity	→	Knowledge	.19	0.09	7.54	<.001
Marital status	→	Knowledge	.32	0.13	15.09	<.001
**Number of children**	** → **	**Knowledge**	**−.05**	**0.03**	**−1.08**	**.282**
Age	→	Opinion	−.48	0.00	−17.36	<.001
**Gender**	** → **	**Opinion**	**-.01**	**0.05**	**−1.32**	**.186**
Education	→	Opinion	−.09	0.05	−2.85	<.001
**Religiosity**	** → **	**Opinion**	**.01**	**0.06**	**0.36**	**.718**
Marital status	→	Opinion	−.17	0.10	−12.85	<.001
Number of children	→	Opinion	−.31	0.02	−12.24	<.001
Knowledge	→	Opinion	.15	0.02	8.09	<.001

Abbreviations: *SE* Standard error

Bolded results are non-significant

**Table 5:** Mediation (indirect) effects analyses

Path					Effect	LL	UL	*P value*
Age	→	Knowledge	→	Opinion	−.13	−.18	−.09	<.001
Gender	→	Knowledge	→	Opinion	−.02	−.03	−.01	<.001
Education	→	Knowledge	→	Opinion	.02	.01	.03	<.001
Religiosity	→	Knowledge	→	Opinion	.03	.02	.04	<.001
Marital status	→	Knowledge	→	Opinion	.05	.03	.07	<.001
Number of children	→	Knowledge	→	Opinion	−.01	−.02	.01	.330

Analyses used bootstrapping (95% bias-corrected, 5,000 resamples). Abbreviations: Effect = standardized indirect effect (predictor → through mediator → criterion). LL = lower limit of the confidence interval; UL = upper limit of the confidence interval

## Discussion

Our study examined the level of knowledge, attitudes, and degree of support for organ donations of porcine origin in Muslim patients. We described the methodological aspects of the study, presented the findings of the survey, and discussed the meaning of our findings and their practical implications.

A full understanding of the beliefs and practices of Muslim traditions will be necessary to fully prepare for implementing clinical trials, i.e., animal organ transplantation. From the Islamic religious perspective, the ethical dimensions of xenotransplantation relate to several aspects. Firstly, similar to many religious traditions, the Islamic tradition is multiple, and many viewpoints can be found on almost any subject. Secondly, there are various moral traditions within Islam, each providing a different ethical view. At present, many patients need kidney and other organ transplants; however, they are not receiving treatment due to the severe shortage of human organ donors (Bokek-Cohen et al., 2023; Padela &Auda, 2020; Qureshi & Padela, 2016).

Xenotransplantation utilizing pig organs may provide the most immediate solution to this problem. The animal closest to human physiology that has demonstrated a high rate of success in clinical trials is the pig (Cooper et al., 2016; Galvao et al., 2023; Lei et al., 2022). Therefore, if pig organs are to be used, it is important to consider the position of religious and ethnic groups on the subject of pig organ transplantation (Babos et al., 2021; Padilla et al., 2020; Qureshi & Padela, 2016; Rabbani, 2010; Randhawa & Neuberger, 2016; Shabana, 2014; Vincent et al., 2011).

From an Islamic legal point of view, treatment based on the use of pig organs and porcine products may, at best, be considered permissible only if there is a Darurah. However, even in cases where the use of porcine products is permissible, the patient is not obligated under Islamic law to undergo the treatment. Furthermore, if the benefits are also accompanied by harmful side effects to the patient, the legal assessment may change to a status of condemnable or forbidden (Bokek-Cohen et al., 2023; Jamaludin, 2012; Shiwani, 2020).

When therapeutic considerations supersede all other considerations, many Islamic jurists rule that the use of pig products and transplants is permitted by Sharia. Nonetheless, some scholars agree that the normative prohibition is canceled when life is in danger. On the other hand, some jurists limit their permission to cases where animals other than pigs are used, based on the idea that the pig is considered intrinsically impure, according to most Sunni scholars. Hence, no part of the pig, such as skin, bones, or organs, may be used by Muslims for medical purposes. Some jurists allow using porcine products in cases of Darurah, which is an extreme necessity (Jamaludin, 2012; Shiwani, 2020).

This is the first comprehensive study that has examined how Muslims perceive the religious permissibility of using organs of porcine origin in medical treatment and their knowledge regarding which medical treatments, if any, should be permitted according to Islamic religious laws and rulings. The present study’s contribution is threefold. First, it is the first comprehensive study that included a wide range of uses of organs derived from pigs, some lifesaving and some not. Secondly, the relatively large number of Muslim participants made it possible to compare the knowledge and attitudes measures of Muslim individuals who belong to the second largest mainstream religious group in the world and who consider the pig an impure animal. Thirdly, the individual’s level of knowledge as to whether their religion forbids or allows this according to Sharia was taken as a variable explaining their position towards the use of pig organs. This was in contrast to previous studies on attitudes towards xenografts or acquiring organs from pigs for the first time. Our findings indicated that the Muslim public is unaware of the fact that the transplantation of life-saving organs of porcine origin is permitted in cases of extreme necessity.

We found that 20.38% of the Muslim respondents answered,"I don’t know"about their knowledge regarding the religiously permissible use of organs for each of the 6 different organs, and 79.62% of respondents either have a low knowledge or expressed uncertainty about the topic. The respondents indicated"I don’t know"in descending order from highest to lowest for the following: skin graft, 17.6% out of 156; cartilage, 16.5% out of 146, pancreas, 15.5% out of 137; kidney, 14.6% out of 129; lungs 13.6% out of 120; and heart 12.6% out of 111. This finding could be associated with the Islamic rulings regarding medical uses of porcine products, such as xenotransplantation that are complex and subject to differing interpretations, as mentioned, of dire necessity, patient distress, and probability of outcome. The study mentions the concept of “Darurah” (necessity), which can permit the use of such products in life-threatening situations. However, these rulings are not universally understood or communicated to the public, leading to a lack of awareness among Muslims about the permissibility of such medical interventions. The lack of clear religious education on medical bioethics in Islam could be a significant reason for the knowledge gap. To improve understanding, we recommend that future studies provide more detailed explanations of these religious concepts and address the lack of clear religious education on medical bioethics in Islam, which could be a significant reason for the knowledge gap.

Cooper and colleagues’ study (2018) primarily centered on the scientific and logistical aspects of xenotransplantation, particularly addressing advancements in using porcine organs for human transplants and overcoming immunological barriers. Cooper and colleagues’ study (2018) considered the broader ethical concerns of xenotransplantation but did not delve deeply into specific religious perspectives, especially within Muslim communities. Our study, on the other hand, specifically examined how the knowledge of Islamic rulings on porcine-derived transplants affects Muslim attitudes toward their use in medical procedures. The findings revealed that many Muslims were unaware that Islamic law permits the use of porcine organs under certain life-threatening conditions. This lack of knowledge correlated with less favorable attitudes towards these transplants, while increased awareness led to more positive attitudes. In summary, while Cooper and colleagues’ study (2018) provided a general ethical framework for xenotransplantation, our study introduces a new dimension by focusing on the religious knowledge within the Muslim community and how it directly influences acceptance or resistance to such medical practices.

The life-saving medical uses of pigs included in our research questionnaire were transplantation of: (1) heart (2) lung (3) kidney (4) pancreas (5) knee cartilage (6) skin. According to the responses as to whether it is permissible to take an organ from a pig, the order of priority in accepting organs from a pig was heart transplant, lung transplant, pancreas transplant, kidney transplant, cartilage transplant, and skin transplant. In addition, the issue of whether porcine organ donations should be permitted was examined. We found that there was a consistency in the order of priority regarding whether it is permissible to take organs from a pig and the use of porcine organs under certain life-threatening conditions.

The history of human organ transplant medicine shows that the main difficulty faced by religious jurists is related to the definition of brain death and taking organs from brain-dead humans. This has caused a crisis of confidence in transplant medicine amongst large groups in the population who are initially more suspicious of advanced medical technologies (Machado, 2007).

Porcine organ transplants are a blessed process that releases those waiting for a transplant from the shortage of organs and the ethical restrictions on donating an organ from a human. The advantages offered by this type of transplantation exceed the issue of using animals for human benefit (Machado, 2007; Shiwani, 2020; Tarabeih et al., 2023).

### Implication for Practice

From the perspective of the Islamic tradition, it must be recognized that XTx raises certain theological issues that should be considered before it is widely introduced. The ideas presented herein suggest that each theological perspective provides the framework for evaluating the possible use of XTx based on the Quran’s call to bring healing to those in need. It may also offer a process for addressing the ethical issues this may pose. Providing full information regarding the available alternative treatments is part of the informed consent process.

Recognizing the patient’s religious beliefs is a very important factor in the interaction between the physician and patient when obtaining informed consent for any medical treatment. Failure to respect religious sensitivities as to the use of organ transplants and biological products, i.e., materials derived from pigs, can have serious medico-legal consequences. The nephrologists, transplant physicians, and transplant coordinators have a weighty responsibility to be aware and sensitive to the patient’s religious background (Curlin et al., 2005; Sattar et al., 2004). This sensitivity is part of respecting patient autonomy and the medical code of ethics (Padilla et al., 2020, 2021; Shiwani, 2020). These situations constitute an ethical challenge for the medical team treating Muslim patients and should be raised in the informed consent process. The medical staff is obligated to provide patients with sufficient information to allow them to make an informed judgment as to their preferred treatment, whether drugs or a porcine organ transplant. This issue may also affect their commitment to the profession and to patients. However, alternatives to pig organs should be considered when possible (Curlin et al., 2005). When no alternative can be substituted, patients, family members, guardians, and even religious leaders should be involved in the decision-making process (Sattar et al., 2004).

Medical councils should consider presenting a separate consent form for transplanting an organ of porcine origin. Besides protecting physicians and patients, this will also improve the level of informed consent as part of good medical practice. Healthcare providers should be made aware of the following: (1) Islamic jurists have widely accepted the use of all animal and human-derived products, specifically in life-threatening cases and when no other alternatives are available. (2) Medical teams should involve Muslim patients in the decision-making process as to their medical care. Patients should be allowed to consult with their religious leaders and enquire whether the animal-derived product is religiously permitted. (3) Informing patients as to their treatment promotes respect for their religious beliefs, which helps promote the therapeutic alliance and therefore, may carry implications for public health.

### Study Limitations

The study employed snowball and convenience sampling, which may not be representative of the entire Muslim population in Israel. This method can introduce biases as it relies on participants to recruit other participants, potentially leading to a non-random sample. The data collection was self-reported via an online questionnaire that can be subject to various biases. These include a social desirability bias, where respondents may answer questions in a manner, they believe is more socially acceptable rather than reflecting their true beliefs. The study’s measures of knowledge and attitudes were based on self-assessment and subjective perceptions. This approach may not accurately capture the depth of understanding and complexity of attitudes regarding the use of medical treatments derived from pigs. The study addresses a highly sensitive topic involving religious beliefs and medical ethics. Participants’ responses may be influenced by their interpretations of religious beliefs, which can vary widely within the Muslim community.

## Conclusion

The attending hospital staff must consider the religious beliefs of all patients and recognize that the patient has the right to receive complete, relevant, and accessible information concerning their treatment. This will empower them to choose the type of healthcare that meets their health, emotional, spiritual, cultural, and religious needs.

Information on the Islamic tradition provided herein will hopefully serve as a source for future discussions amongst the principals involved in XTx programs and help promote theological understanding and respect for the impact they may have on the individual patient. If XTx is to gain full public acceptance and support, the medical profession must recognize that for some, the decision to seriously consider pig organ transplantation will never be possible due to their personal religious beliefs.

Our findings demonstrated that Muslims were unaware of what is permitted by Islam. It is crucial to obtain informed consent for using medical products of animal or human origin for followers of Islam and other religions with similar prohibitions since they may oppose such treatment. We believe that the findings of our study will be used as an evidence-based source for deliberations among physicians, religious leaders, and theologians to promote knowledge in members of their religions. No less importantly, they may enrich the cultural competence of medical and nursing staff with respect for the autonomy of any patient.
